# Serum 25(OH)D and Cognition: A Narrative Review of Current Evidence

**DOI:** 10.3390/nu11040729

**Published:** 2019-03-29

**Authors:** Mary A. Byrn, Patricia M. Sheean

**Affiliations:** School of Nursing, Loyola University Chicago, 1032 W Sheridan Rd, Chicago, IL 60660, USA; psheean1@luc.edu

**Keywords:** vitamin D, cognition, dementia, obesity

## Abstract

The effect of low serum 25(OH)D on cognitive function is difficult to determine owing to the many factors that can influence these relationships (e.g., measurements, study design, and obesity). The primary purpose of this review was to synthesize the current evidence on the association between serum 25(OH)D and cognition giving special consideration to specific influential factors. A search was conducted in PubMed for studies published between 2010 and 2018 using terms related to serum 25(OH)D and cognition. Only studies that used liquid chromatography tandem-mass spectrometry (LC-MS) were included, since this is considered the ‘gold standard method’, to measure serum 25(OH)D. Of the 70 articles evaluated, 13 met all inclusion criteria for this review. The majority of the observational and longitudinal studies demonstrate a significant association between low serum 25(OH)D and compromised cognition. However, two randomized controlled trials showed inconsistent results on the impact of vitamin D supplementation on cognitive function. The varied methodologies for ascertaining cognition and the inclusion or exclusion of confounding variables (e.g., obesity, sunlight exposure) in the statistical analyses make drawing conclusions on the association between serum 25(OH)D and cognitive functioning inherently difficult. Despite the known higher occurrence of serum 25(OH) deficiency among minority populations, the majority of studies were conducted in with White participants. In order to more clearly discern the relationship between serum 25(OH)D and cognitive functioning, future studies should target more diverse study populations and utilize comprehensive measures to reliably capture cognition, as well as important known determinants of serum 25(OH)D.

## 1. Introduction

Dementia is a general term that describes a decline in mental ability severe enough to interfere with daily life. There is no cure for dementia; thus, it is imperative to identify strategies to prevent or slow the progression of this debilitating disease. Alzheimer’s disease—the most common type of dementia—constitutes 60 to 70 percent of all dementia cases [1]. It is estimated that 50 million people worldwide are currently inflicted with dementia [1]. Mild cognitive impairment is considered a precursor to its Alzheimer’s disease and is more common, effecting 15–20% of people age 65 or older [2].

Vitamin D, also referred to as serum 25(OH)D, reflects a group of fat-soluble steroids best known for increasing the intestinal absorption of minerals, specifically calcium, magnesium, and phosphate. The impact of serum 25(OH)D on skeletal diseases, such as rickets, has been well documented. Interestingly, there is growing appreciation for the role of vitamin D in cognition and potentially Alzheimer’s disease [3]. Vitamin D metabolites, their related enzymes, and vitamin D receptors (VDR) have been found in the brain [3,4]. Specifically, they have been found in neurons and glial cells of the hippocampus, hypothalamus, cortex, and subcortex [4]. These are also areas in the brain known to be related to learning and memory. Alzheimer’s disease is a neurodegenerative disease marked by consistent decline in cognition. The cause of Alzheimer’s disease is unknown, however the possible association between Alzheimer’s disease and vitamin D was first proposed in 1992 when decreased VDR levels in the hippocampus of patients with Alzheimer’s disease were reported [3]. More recently, there have been genetic studies which document polymorphisms in the VDR which are related to an increase risk in poor cognition or development of Alzheimer’s disease [3]. The Institute of Medicine (IOM) defines vitamin D insufficiency as a serum 25(OH)D level ≤20 ng/mL, based on the implication for bone health [5]. However, the Endocrine Society supports the view that a serum 25(OH)D ≤30 ng/mL is insufficient [6]. Applying the levels of the IOM (≤20 ng/mL), current estimates support that 39% of healthy adults in the US are vitamin D deficient [7]. When using the values set forth by the Endocrine Society (≤30 ng/mL), the number US adults with vitamin D deficiency increases to 64% [7]. Modifiable risk factors for vitamin D deficiency include decreased sunlight exposure, reduced dietary vitamin D intake, and obesity [8].

Competitive binding methods, high performance liquid chromatography (HPLC), and radioimmunoassay (RIA) are the most common methods used to determine serum 25(OH)D, yet each has inherent variation and drift [9] making it difficult to compare findings across studies. To help increase precision and to reduce the impact of metabolites on vitamin D measures, liquid chromatography tandem-mass spectrometry (LC-MS) is considered the ‘gold standard’ measurement for both 25(OH)D_2_ and 25(OH)D_3_. The general purpose of this narrative review is to synthesize the current evidence on vitamin D and cognition in adult populations, taking into account assay methodology and other known determinants. First, to address the acknowledged differences by laboratory technique, this review will only include studies that employ the LC-MS methodology for serum 25(OH) determination. Second, we will assess if each study considered important confounders on the relationship between serum 25(OH)D and cognition, recognizing the role of obesity, sunlight exposure, dietary intake, and supplementation.

## 2. Materials and Methods

A comprehensive literature search was conducted using the PubMed database with search terms of the Medical Subject Headings (MeSH), including “Vitamin D”, “Calcitriol”, Cognition Disorders”, and “Cognition”. The search was further limited to include humans, articles published in English, and studies with participants older than 19 years of age. All relevant articles were initially assessed for eligibility. Studies were included in this review if they met the following criteria; published between 2010 and 2018, included adults participants (defined as 19 years of age and older), reported serum vitamin D or 25(OH)D, used the LC-MS methodology to determine serum 25(OH)D, published in a peer-reviewed journal, and an objective measure of cognition was used. Studies conducted prior to the year of 2010 were excluded based on the variability in methods and the expert recommendation to use the LC-MS methodology starting in 2010 [10]. Although there are over 50 metabolites of vitamin D, serum 25(OH)D, which is comprised of 25(OH)D_3_ and 25(OH)D_2_, is considered the best marker of vitamin D nutriture [11].

## 3. Results

Originally, 70 articles were evaluated and 13 met all inclusion criteria for this review. Of these, three studies were observational and eight were longitudinal. Participants in these studies were from five countries (Netherlands, United States, Korea, Canada, and Sweden), largely White with an average age of 71 years old. Two studies were randomized trials, including participants from Canada and Australia with a mean age of 44 years. This review is organized based on study design and all studies are detailed in Table 1, Table 2 and Table 3.

### 3.1. Observational Studies

Three studies in this review utilized a cross-sectional design (see Table 1). Brouwer-Brolsma et al. (2013) [12] performed a cross-sectional analysis using a sample of 127 Dutch participants 65 years and older who were enrolled in the ProMuscle Study. Brouwer-Brolsma et al. (2013) [12] reported a significant positive association between cognition tasks involving executive functioning, measured by the Verbal fluency test and Reaction Time Task, and serum 25(OH)D. Brouwer-Brolsma et al. (2015) [13] completed another cross-sectional analyses using data from the B-PROOF study, which was a randomized control trial investigating vitamin B12 and folic acid supplementation in participants 65 years in older living in the Netherlands. Participants were eligible for this analysis if they had a serum 25(OH)D measure (*n* = 2857). A battery of cognitive exams was used to assess four cognitive domains. Out of the 2857 participants, 846 elderly participants had completed the measure for attention and working memory (The Digit Span forward and Backward test) and participants with a 25(OH)D level greater than 70 nmol had a 50% lower chance of scoring in the lowest 10% of cognitive performance. Both analyses were completed controlling for age, sex, BMI, education, smoking, alcohol consumption, habitual physical activity, and season of blood sampling [12,13]. However, diet intake and vitamin D supplementation were not accounted for in the analysis. Milman et al. (2014) [14] recruited 253 Ashkenazi Jewish participants who were all 95 years or older and living independently from the Northeastern United States. Milman et al. (2014) [14] reported that insufficient serum 25(OH)D levels were significantly related to global cognitive impairment. Vitamin D supplementation and diet intake were not controlled for in the analysis. Insufficient 25(OH)D levels (<30 ng/mL) were found in 71.8% of participants with cognitive impairment compared to 57.7% participants with normal cognitive function [14].

Collectively, these three studies found a significant association between 25(OH)D and cognition [10,11,12], providing strong evidence that as serum vitamin D decreases cognitive functioning also decreases. However, when considering the factors that increase the risk of low vitamin D and worse cognition, it is difficult to determine the temporality of events. People with worsening cognition have many risk factors for vitamin D deficiency including: decreased ability for the skin to produce vitamin D related to skin aging, longer hours indoors resulting in lower sun exposure, and living in residential care. Therefore, reverse causality is plausible in this situation and cannot be ruled out based on the cross-sectional design.

### 3.2. Longitudinal Studies

Eight of the studies included in this review utilized a longitudinal design (see Table 2). Slinin et al. (2012) [15] conducted a prospective cohort study in a cohort of women who were enrolled in The Study of Osteoporotic Fractures Study. The Study of Osteoporotic Fractures Study originally recruited 9704 Caucasian women aged 65 years and older between 1986 and 1988 from Maryland, Minnesota, Pennsylvania, and Oregon. The Year 6 visit in the Study of Osteoporotic Fractures Study was the baseline visit for the current analysis which resulted in a sample of 6257 women. Cognition was measured using the modified Mini-Mental State Examination (mMMSE) and Trail Making Test Part B. Findings showed that women with a serum level of 25 (OH) D <10 ng/mL at baseline was associated with worse cognition, as measured by mMMSE after four years and controlling for several confounders. However, diet intake of vitamin D and vitamin D supplementation were not accounted for in the analysis. The association between low baseline 25(OH)D and worse cognition four years later was significant, even though women without dementia were not included in the analysis and women who were lost to follow-up were more likely to be older, have lower 25(OH)D levels and worse cognition at baseline [15].

Moon et al. (2015) [16] examined the association between the development of mild cognitive impairment and low serum 25(OH)D. This prospective study was completed with a sample of elderly Korean men and women as part of the Korean Longitudinal Study on Health and Aging. After excluding participants with dementia at the baseline measure in 2005–2006 (*n* = 7), 405 participants were followed for five years (final measure completed in 2010–2011). Using the Korean version of the Mini International Neuropsychiatric Interview and the Korean version of the Consortium to Establish a Registry for Alzheimer’s Disease Clinical Assessment Battery, 67 participants had progression to mild cognitive impairment or dementia over the five years. Also, participants with severely deficient 25(OH)D levels (<25 nmol) and a MMSE score of less than 27 at baseline were more likely to develop mild cognitive impairment or dementia over the five years. No dietary intake or vitamin D supplementation was included in the analysis [16].

Pettersen et al. (2014) [17] studied 32 participants from Canada to assess the association between cognition and serum 25(OH)D levels and how seasonal fluctuations impact cognitive function. Thirty-two healthy participants completed baseline measures in the summer; however, 19 participants completed follow-up measures in winter and were included in the longitudinal analysis. These authors found that participants with more than a 15 nmol decline in serum 25(OH)D between summer and winter months had significantly more decline in working memory and executive functioning, as measured by the one touch Stockings of Cambridge (otS) task [17]. Findings from this small study support a seasonal impact on both, serum 25(OH)D and cognitive function.

Littlejohns et al. (2014) [18] and Kuzma et al. (2016) [19] both used populations from the US Cardiovascular Health Study (CHS). Participants in the CHS were recruited from North Carolina, California, Washington, and Pennsylvania. The CHS included 5201 participants that were initially recruited in 1989–1990 and 687 African-American participants that were recruited in 1992–1993. In the CHS study, serum 25(OH)D levels were collected in 2312 participants that were free from cardiovascular disease during the annual data collection visit in 1992–1993. Littlejohns et al. (2014) [18] excluded participants from their study if they did not have a serum 25(OH)D level, had prevalent cardiovascular disease or stroke, or undocumented dementia status. Participants with dementia at the time that the vitamin D was collected were excluded from the main analysis but were included in the prospective analysis. Participants who were lost to follow-up were older, non-White, and had lower serum 25(OH)D levels. The final sample for the prospective analysis consisted of 1658 participants. Dementia and Alzheimer’s disease were assessed using the National Institute of Neurological and Communicative Diseases and Stroke/Alzheimer’s Disease and Related Disorders Association (NINCDS-ADRDA) criteria. Those with severely deficient 25 (OH) D levels (<25 nmol) were found to be 2.2 times more likely (95% CI: 1.02–4.83) to develop Alzheimer’s disease (mean follow-up of 5.6 years) compared to participants with sufficient 25 (OH) D levels (≥50 nmol/L) [18]. Diet intake of vitamin D and vitamin D supplementation were not controlled for during the analysis. Kuzma et al. (2016) [19] employed a different methodology using this same cohort of participants. Specifically, in the Kuzma study, participants were excluded from the CHS sample who had a diagnosis of dementia (based NINCDS-ADRDA criteria), were aged 65 years and older, had a baseline measure of 25(OH)D, and two or more cognitive assessments. Participants who were determined to have substantial cognitive decline (defined as ≥1 SD decrease greater than the mean change score from baseline to the final assessment) were also excluded from the final analysis. The final sample consisted of 1291 participants. Kuzma et al. (2016) [19] found that only the participants who were severely deficient (<25 nmol/L) had a significant decline in visual memory (measured by Benton Visual Retention Test) and global cognition (measured by Modified Mini- Mental State Exam). The analysis was not completed controlling for diet intake or vitamin D supplementation [19].

Kueider et al. (2016) [20] included 1207 participants from the Baltimore Longitudinal Study of Aging (BLSA) to study if serum 25(OH)D levels are associated with cognition. The BLSA began in 1958 and enrolled adult participants from the community to participate in the prospective cohort study. Kueider et al. (2016) [20] included the participants that did not develop dementia or mild cognitive impairment at any point during the study. Participants were assessed every two years starting in 2003 and followed for an average of 10.4 years. Mendelian randomization analyses were used and a causal relationship between lower serum 25(OH)D and worse executive functioning (measured by clock-drawing to command test) and psychomotor speed (measured by Purdue Pegboard) were reported [20]. Information on diet intake of vitamin D or vitamin D supplementation was not accounted for in the analysis.

Two additional longitudinal studies reported null findings [21,22]. Olsson et al. (2017) [21], studied the relationship between plasma 25(OH)D and the risk of dementia. Participants were from the Uppsala Longitudinal Study of Adult Men that was completed in Uppsala, Sweden. For this study, participants were excluded if they had a diagnosis of dementia or stroke with aphasia at baseline or developed these diagnoses within two years. The sample included 1182 participants at baseline, however only 408 men completed the follow-up measure 12 years later. A medical diagnosis of Alzheimer’s disease and the MMSE were used to measure cognition. The insignificant findings may be due to high baseline levels of serum 25(OH)D (68.4 nmol), where only 15.5% of the sample had low serum 25(OH)D levels (less than 50 nmol) at baseline and the high attrition rate [21].

Schneider et al. (2014) [22] had a large baseline sample (*n* = 1652) of middle aged participants (52% White, 48% Black, and 60% female) who were enrolled in the Atherosclerosis Risk in Communities (ARIC) Brain MRI Study. The ARIC study is an ongoing prospective cohort study that began in with baseline measures between 1987 and 1989. Schneider et al. (2014) [22] excluded participants whom had insufficient serum for 25(OH)D, had a history of stroke or transient ischemic attack, or a diagnosis of dementia using ICD-9 codes, and those who were missing cognitive measures. Cognitive functioning was measured at three visits occurring between (1) 1993 and 1995, (2) 1996 and 1998, and (3) 2004 and 2006. The delayed word recall test (DWRT), digital symbol substitution test (DSST), and the word fluency test (WFT) were used to measure cognitive functioning. Serum 25(OH)D was measured in 2012 using serum from samples collected between 1993 and 1995. No significant association between low serum 25(OH)D and any cognitive measure (DWRT, DSST, or WFT) was found [22]. There were 145 participants hospitalized with an ICD-9 code for dementia over the median of 16.6 years of follow-up, however there was no significant relationship with serum 25(OH)D and development of dementia. In summary, out of the eight longitudinal studies, six reported a significant relationship between low serum vitamin D at the start of the study and compromised cognition at the follow-up data collection point. Two studies reported null results. While these longitudinal observational studies are limited by potential confounding events between assessment points (e.g., acute medical events, sunshine exposure, vitamin D supplement discontinuation, or initiation), they provide important foundational information regarding the plausibility of the exposure disease relationship concerning serum 25(OH)D and cognition.

### 3.3. Randomized Control Trials

Two studies included in the review were completed using a randomized controlled trial design (see Table 3). Dean et al. (2011) [23] enrolled 128 healthy, young adults from Queensland, Australia. Participants were randomized to receive 5000 IU of Vitamin D (cholecalciferol) daily or placebo (lactose) for six weeks. Assessments were completed at a baseline visit and after 6 weeks of placebo or vitamin D supplementation. Cognition was assessed using the N-Back task to measure working memory, the stop-signal task to measure response inhibition, and set shifting task to measure cognitive flexibility. Participants had normal serum 25 (OH) D levels at baseline with an average of 76.6 nmol/L (SD 19.9), and only ten participants have a baseline serum 25(OH)D less than 50 nmol/L, indicating insufficient levels. No significant improvement was found in cognitive functioning in the group receiving vitamin D supplementation compared to the placebo group on any of the cognitive measures [23]. The analysis was completed without controlling for any covariates and which could have an impact on the validity of the findings.

Pettersen et al. (2017) [24] included 82 healthy adults from British Columbia, Canada in a randomized control trial to investigate if vitamin D supplementation would improve cognitive functioning. Participants with baseline 25(OH)D levels ≤100 nmol were randomized to either receive a high dose vitamin D_3_ (cholecalciferol) of 4000 IU/day or a low dose of 400 IU/day for 18 weeks. Cognitive functioning was measured using the Symbol Digit Modalities Test (SDMT) to assess information processing speed, verbal fluency to assess executive functioning, digit span forward and backward to assess attention/working memory, and the CANTAB^®^ battery, which also includes measures for verbal learning, verbal recognition memory, nonverbal learning, pattern recognition memory, paired associates learning, working memory, and executive functioning. When examining baseline characteristics (e.g., age, sex, and education level), no differences between participants who received the high dose and low dose supplement at baseline were reported. After 18 weeks of supplementation, serum 25(OH)D levels increased significantly more in the high vs. low dose group (130 nmol/L vs. 85.9 nmol/L, respectively). There were no patients with insufficient 25(OH)D levels in the high dose group and 22.5% of the participants in the low dose groups remained insufficient (<75 nmol/L). Participants taking the high dose supplement significantly improved on two cognitive tests for visual memory compared to the low dose group. However, after controlling for potential confounders, the improvements on pattern recognition memory task and paired associates learning task were insignificant [24]. The analysis did not control for diet intake or vitamin D supplementation. Participants in the low dose supplement group improved significantly on the recognition component of the verbal memory task compared to participants in the high dose group. Participants with an insufficient serum 25(OH)D level at baseline (<75 nmol/L) and assigned to the high dose group, improved significantly on the pattern recognition memory task compared to those with insufficient serum 25(OH)D levels in the low dose group.

## 4. Discussion

This article summarizes the literature to date on the associations between serum 25(OH)D and cognition in adults, controlling for the great variability in various assay methodology by limiting studies to those that use the recommended LC-MS assay for serum 25(OH)D determination. This topic is particularly relevant given the burgeoning aging population and the growing awareness between serum 25(OH)D and a variety of health outcomes. Reviews of this type are often difficult to perform, as the inherent purpose is to simplify findings from studies that possess highly variable research hypotheses, study designs and objectives. As reflected in Table 1, Table 2, and Table 3, studies reported a variety of aspects of cognition including: executive functioning, attention, working memory, information processing speed, verbal learning, and visual memory. Despite these differences, common themes emerged regarding the results and limitations (see Table 4).

First, although the methodology to measure serum 25(OH)D was consistent among all studies, the approaches to measure cognition were quite varied making it intrinsically difficult to draw definitive conclusions regarding the associations between serum 25(OH)D and cognition. Six of the thirteen studies used the MMSE to gauge cognitive function [12,13,14,16,20,21]; only two of these studies reported significant MMSE findings [14,15]. Additionally, two studies used a modified version of the MMSE (mMMSE) [15,19], so direct comparisons to other studies is limited. The mMMSE includes four additional test items and is designed to measure a broader variety of cognitive function thereby improving the instruments ability to detect cognitive impairment [25] While most of the studies used the MMSE to measure the outcome variable of global cognition, the study by Moon et al. (2015) [16] was slightly different from others. To ascertain a diagnosis of MCI or dementia, these investigators administered the MMSE, as a baseline measure of cognition. However, to measure cognition longitudinally the Korean version of the Consortium to Establish a Registry for Alzheimer’s Disease Clinical Assessment Battery and the Korean version of the Mini International Neuropsychiatric Interview were used at baseline and follow-up visits. This approach allowed the authors to detect definitive diagnostic changes in cognition over time versus an overall global cognitive functioning ascertained when just the MMSE is used at one time point. Interestingly, seven of the studies that used the MMSE also administered at least one other instrument to measure cognition. Although only two studies reported significant findings with the MMSE, four studies reported significant findings when another instrumentation was used to measure cognition. For example, Brouwer-Brolsma et al. (2013) [12] and Brouwer-Brolsma et al. (2015) [13] had similar measures for cognition and both studies used a cognitive battery including digit span forward and backward test, Trail Making Tests A and B, Stroop Color–Word Test, the Verbal Fluency test, and the MMSE. Brouwer-Brolsma et al. (2015) [13] reported significant findings on Digit Span forward and backward and Brouwer-Brolsma et al. (2013) [12] reported significant findings on the Reaction Time Test, which was not used in Brouwer-Brolsma et al. (2015) [13] study. While these studies employed similar tools in similar populations, these inconsistencies may signify limitations in the instrumentation potentially diminishing or masking the links between cognitive functioning and serum 25(OH)D status.

To this end, Kueider et al. (2016) [20] and Milman et al. (2014) [14] also both used the MMSE to measure global cognition and the clock-drawing test to measure executive functioning; another aspect of cognition. The Clock-drawing Test has two parts as options when being administered. The first option is instructing the patient to draw a clock with the hands of the clock being set at ten past eleven, and the second part is where the patient is instructed to copy a picture of a clock with the hands being set at ten past eleven. Kueider et al. (2016) [20] reported significant findings on the command option of the clock-drawing test, but not the MMSE while Milman et al. (2014) [14] reported significant findings on the MMSE and the copy clock-drawing test. Perhaps the discrepancies in significant findings with the use of the MMSE relates to inherent restrictions of this measurement tool. Although the MMSE is widely used in longitudinal studies to track changes in cognition over time, ceiling/floor effects can lower the ability of the tool to measure true changes in cognition throughout the duration of a study [26], which would create a significant barrier to linking changes in cognition with serum 25(OH)D. The maximum MMSE score of 30 has been found to be easily obtained by participants with high levels of education and, likewise, the minimum score may be easily obtained by people with severely impaired cognition, making it impossible to detect any improvement or decline in cognition over time in these participants [26]. Another limitation of the MMSE is the varied curvilinearity or sensitivity to change [26]. The psychometric property of curvilinearity reflects the concept that a change in the measurement tool many not represent the same intensity of cognitive change at all times [27]. Meaning, a 1-point change in the MMSE score from baseline to follow-up can have different meanings simply based on the initial score. These measurement difficulties with the MMSE can be more profound when using the tool to assess for changes in cognition in a sample of people that have varied levels of cognitive functioning. Taken together, these methodological challenges of quantifying cognition changes over time may have contributed to the differing documented significant findings on the impact of serum 25(OH)D on cognition in the studies reviewed. To combat these measurement problems, Philipps et al. (2014) [26] recommend a normalizing transformation analysis when using the MMSE to detect cognitive change and none of the studies reviewed performed the analysis in this manner. Given these challenges when using the MMSE to measure cognition over time, future research should use a battery of cognitive tests that are more sensitive to detecting changes in cognition over time. The Cambridge Neuropsychological Test Automated Battery (CANTAB) is an example of a battery that has been found to be reliable for use in patients with both mild cognitive impairment and Alzheimer’s disease [28], and should be considered in studies going forward.

Second, obesity has long been considered a risk factor for vitamin D deficiency. Results from several large trials, including the National Health and Nutrition Examination Survey (NHANES) [29] and the Framingham Heart Study [30], have demonstrated an inverse relationship between serum 25(OH)D and BMI. That is, as BMI increases, serum 25(OH)D decreases. This concept is not new, but remains poorly understood. Cipriani et al. [31] proposes at least three mechanisms to explain this inverse relationship: (1) serum (OH)D moves out of circulation and is sequestered into the adipose tissue, (2) serum 25(OH)D potentially undergoes alterations in metabolism from hepatic steatosis; or (3) serum 25(OH)D is lowered in the blood due to the inhibitory effects of adipokines [31]. Further, low levels of serum 25(OH)D have been postulated to simply be a consequence of obesity-associated volumetric hemodilution [32]. Regardless of the etiology, the majority of studies included in this review did attempt to address the impact of obesity on vitamin D status by controlling for BMI during the analyses. Only three studies did not address obesity [17,23,24]. While BMI has been used a surrogate marker of adiposity for some time [33,34], a recent systematic review and meta-analyses of 31,968 healthy participants revealed that BMI fails to detect half of the people with excess adiposity [35]. Thus, its application as a surrogate marker for adiposity is questionable, underscoring the need to include more comprehensive methods to exam body composition going forward. This is especially relevant in studies addressing cognition, where study populations are typically older individuals and total adiposity increases while lean mass decreases. To more precisely capture body composition, the inclusion of dual-energy X-ray absorptiometry is recommended for future studies.

Third, sun exposure and dietary supplementation and, to a lesser extent, dietary intake are all major determinants of serum 25(OH)D levels [6]. Only six of the thirteen studies attempted to control for sun exposure and did so by including season of blood draw as a covariate in the analysis [12,13,15,18,19,21,22]. Although many older people spend a considerable amount of time indoors, it would be more methodically sound to gather data directly on time spent outdoors, especially during the summer months when the UV index is higher and translates to higher cutaneous conversion of vitamin D. Glanz et al. [36] developed a tool in 2008 to quantify sunlight exposure, sun protective practices, and skin pigmentation to address this concern in behavioral and intervention trials [36]. Further, none of the studies reporting significant associations between serum 25(OH)D and cognition collected information on dietary intake of vitamin D; however, three studies did gather data on vitamin D supplementation and control for this in the analysis [15,21,22]. Interestingly, two of the studies that measured vitamin D supplementation did not report a significant association between serum 25(OH)D and cognition [21,22]. Therefore, the lack of information of vitamin D intake through diet or, more importantly, dietary supplementation is worrisome and needs stronger attention going forward. Rather than control for season of blood draw or ignore the important contributions of diet and supplementation, future studies should better quantify sunlight exposure and dietary intake, specifically focusing on vitamin D supplements and occult sources of vitamin D (e.g., combination calcium and vitamin D supplements).

Fourth, vitamin D deficiency is more prevalent among minority populations [37,38]. However, the populations included in this review were predominantly White. Two studies included race/ethnicity in their demographic explanations of the study populations, had exclusively White study participants [15,20], while five studies did not specify information regarding racial origins of the study population. Brouwer-Brolsma et al. (2013 & 2015) [12,13] recruited Dutch participants, Olsson et al. (2017) [21] included Swedish participants, the investigation by Milman et al. (2014) [14] was comprised of Ashkenazi Jewish participants, and Pettersen et al. (2017) [24] targeted participants from Northern British Columbia, Canada. Hence, we can speculate that these were largely White participants. Persons of Hispanic origin were not represented in any of the aforementioned investigations. Given that both vitamin D deficiency and cognitive function are more prevalent in minority populations, this lack of diversity decreases the ability to generalize findings and determine the true implications of sufficient serum 25(OH)D levels on cognitive function.

## 5. Conclusions

Although all the studies meeting our inclusion criteria used the most accurate measure to determine serum 25(OH)D levels, our ability to make definitive conclusions regarding 25(OH)D and cognition remains hampered by several factors. We hypothesize the reason for the difficulty in concluding the causal relationship between low serum 25(OH)D and cognition is the variation in study design, sample size, sample characteristics (e.g., age and race), and measures for cognition employed. First, observational study designs predominated over others; thus, determining the cause-and-effect relationship between serum 25(OH)D levels and cognition is simply not possible and the likelihood of reverse causality cannot be ruled out. Future studies should consider other study designs to efficiently and effectively evaluate this relationship (see Table 5). While a randomized controlled trial would be considered methodologically ideal, the length of time (e.g., decades) needed to determine changes in cognition may be financially impractical. A potential design consideration may include recruiting ‘high risk’ populations. Several gene mutations have been discovered in the last decade that can strongly predict early onset Alzheimer’s disease [39]. Recruiting individuals with this genetic predisposition who possess low serum 25(OH)D levels presents an ideal opportunity to test the impact of vitamin D supplementation on cognitive decline. Rather than waiting decades to observe the outcome of interest, cognitive decline occurs more readily providing an efficient design in these vulnerable individuals, for whom the benefits could be quite tangible. In spite of this advantageous study design, determining the amount of vitamin D supplementation to provide remains controversial. Currently, the Institute of Medicine recommends no more than 4000 IU/day of Vitamin D_2_ or D_3_, reflecting the tolerable upper intake level [40]. However, recommendations of up to 6000 IU daily of vitamin D_2_ or D_3_ (or 50,000 IUs weekly for eight weeks) have been suggested [6]. Further, as previously mentioned, these two groups are not uniform in the cut-points used to define ‘deficiency’ (<20 ng/mL vs. <30 ng/mL, respectively). When considering the benign side effects of vitamin D supplementation and potential issues with daily pill compliance, recommendations of 6000 IUs per day and serum targets greater than >30 ng/mL seem practical and safe. Second, the validity of the instrumentation to discern a true relationship between serum 25(OH)D and cognition is concerning. Considerable efforts are needed to identify reliable and comprehensive tools to consistently measure cognition in a multitude of ways. The population that has been mostly studied is older in age, making generalizations on the impact of vitamin D on cognition to younger populations problematic. Finally, the populations who have been studied lack racial/ethnic diversity and, as such, it is difficult to generalize these findings to the populations that are most at risk for low serum 25(OH)D and cognitive dysfunction. Future investigations should include several tools to measures cognition and target Black and Hispanic participants. These measures will help broaden our knowledge and understanding of serum 25(OH)D deficiency and cognitive functioning, especially among vulnerable populations.

## Figures and Tables

**Table 1:** Observational studies examining serum 25(OH)D and cognitive function.

Citation	Sample (size, key characteristics)	Study Design	Cognition Aspect/Measures		Covariates	Outcomes
Brouwer-Brolsma et al. (2013) [12]	127 frail or prefrail Dutch elderly, mean age 79 years, SD 7.6	Cross-sectional	Global Cognition	MMSE	Age, sex, BMI, education, smoking, alcohol consumption, habitual physical activity, and season of blood sampling	Significant positive association between executive functioning and serum 25(OH)D (β = 0.007, *p* = 0.01). For every 11 nmol increase in serum 25(OH)D, 1 more work was memorized on the Word Learning Test.
			Episodic Memory	Word Learning Test direct recall, delayed recall, and recognition		
			Attention and working memory	Digit Span forward and backward test		
			Information processing speed and concept shifting interference	Trail Making Test A and B		
			Selective attention	Stroop Color–Word Test		
			Executive Functioning	Verbal fluency and Reaction Test		
Brouwer-Brolsma et al. (2015) [13]	2857 Dutch participants, 59% male with an average age of 72.5 years.	Cross-sectional	Global Cognition	MMSE	Age, sex, BMI, education, smoking, alcohol consumption, habitual physical activity, and season of blood sampling	Significant association between higher serum 25(OH)D and attention and working memory (PR: 0.50, 95% CI 0.29–0.84)
			Immediate and delayed recall	Rey Auditory Verbal Learning Test		
			Attention and working memory	Digit span forward and backward		
			Information processing speed	Trail Making Test part A and Symbol Digit Modalities		
			Executive functioning (concept shifting interference	Trailing Making Test part B		
			Executive functioning (selective attention)	Stroop Color–Word test		
			Executive functioning	Letter fluency		
Milman et al. (2014) [14]	253 Ashkenazi Jewish with exceptional longevity, median age 97 years	Cross-sectional	Global Cognition	MMSE	Age, sex, BMI, education, history of tobacco use, depression, HDL cholesterol levels, and presence of ≥2 medical comorbidities	Insufficient serum 25(OH)D levels significantly associated with lower global cognition (OR 3.2, 95% CI 1.1–9.29, *p* = 0.03) and CDT (OR 8.96, 95% CI 1.08–74.69, *p* = 0.04)
			Executive functioning	Clock-drawing test		

Abbreviations: MMSE (Mini-Mental State Exam); BMI (Body Mass Index); HDL (High-density lipoprotein).

**Table 2:** Longitudinal studies examining serum 25(OH)D and cognitive function.

Citation	Sample (size, key characteristics)	Study Design	Cognition Aspect/Measures		Covariates	Outcomes
Slinin et al. (2012) [15]	7257 Caucasian women over the age of 65 years, (mean age 76.6, SD 4.7)	Longitudinal	Global Cognition	mMMSE	Clinic site, season, age, years of education, self-reported health status, instrumental activity of daily living impairments, smoking status at baseline, body mass index, history of hypertension, history of diabetes and depression, baseline cognitive function, walking for exercise, and baseline vitamin D supplementation	Low serum 25(OH)D levels were associated with worse cognition (OR 1.60, 95% CI: 1.05–2.42) and more cognitive decline (OR 1.58, 95% CI: 1.12–2.22)
			Executive functioning	Trail Making Test Part B		
Moon et al. (2015) [16]	405 elderly Korean participants with a mean age of 72.5 years (SD 7.0)	Longitudinal	Global Cognition	MMSE	Age, sex, education duration, BMI, baseline MMSE, exercise level, GDS-K and CIRS scores, smoking habit, alcohol intake and the presence of hypertension, diabetes mellitus and stroke history	Participants with severely deficient 25(OH)D levels and poor cognition at baseline were significantly more likely to develop dementia over 5 years (HR 4.66, 95% CI 1.46–14.88 *p* = 0.009)
			Alzheimer’s Disease Diagnosis	Korean version of the Consortium to Establish a Registry for Alzheimer’s Disease Clinical Assessment Battery and the Korean version of the Mini International Neuropsychiatric Interview Lexical Fluency Test Digit Span Test		
Pettersen et al. (2014) [17]	32 participants, mean age of 52 years (SD 16), 72% female, 69% Caucasian	Longitudinal	Information processing speed	Symbol Digit Modalities Test	Age, education, sex	Participants with insufficient serum 25(OH)D levels had significantly lower working memory (M 5.8, SD = 2) compared to those who were sufficient (M = 7.9, SD = 2) (*p* = 0.018) and participants with the most decline in serum 25(OH)D from summer to winter showed more decline in working memory and executive functioning (M = 0.50, SD = 1.9 vs. M = −2.11, SD = 2.6 *p* = 0.01)
			Executive functioning	Phonemic fluency and One-Touch Stockings of Cambridge		
			Attention and working memory	Digit Span forward and backward		
			Learning/Memory	Verbal Recognition Memory, Pattern recognition, and Paired Associate Learning		
			Working Memory	Spatial Working Memory		
Littlejohns et al. (2014) [18]	1658, mean age 73.6 years, 69% female, 88% White	Longitudinal	Dementia and Alzheimer’s Disease Diagnosis	National Institute of Neurological and Communicative Diseases and Stroke/Alzheimer’s Disease and Related Disorders Association (NINCDS-ADRDA) criteria	Age, season, education status, sex, BMI, smoking, alcohol consumption, depressive symptoms	Severe serum 25(OH)D deficiency HR: 2.22, 95% CI: 1.02–4.83) and deficient serum 25(OH)D 1.96, 95% CI 1.06–2.69) was found to significantly increase the risk of developing all cause dementia and Alzheimer’s disease
Kuzma et al. (2016) [19]	1291, average age 72 years, 68% female, 90% White	Longitudinal	Global cognition	3MSE	Age, season of blood draw, education, gender, income, BMI, smoking, alcohol consumption, depressive symptoms, gait impairment	Participants with severely deficient 25(OH)D levels had significant decline in visual memory (−0.10 SD 95% CI:−0.19 to −0.02, *p* = 0.02) and global cognition (RR 1.73, 95% CI 1.22–2.45, *p* = 0.007) compared to those with sufficient levels.
			Visual Memory	Benton Visual Retention Test		
Kueider et al. (2016) [20]	1207, average. age 52.6 years (SD 16.0), All White, 49.8% male	Longitudinal	Global cognition	MMSE	Age, sex, years of education, significant depressive symptoms, BMI, and APOE ε4 status	Significant association reported between low serum 25(OH)D and worse executive functioning on Clock-drawing (3:25 β = 0.05; 95% CI 0.01–0.08, *p* = 0.002 test for endogeneity *p* = 0.001; 11.10 β = 0.03; 95% CI 0.006, 0.06 *p* = 0.02; test for endogeneity *p* = 0.03) and on the Trail Making Test-Part B (β = 0.04; 95% CI 0.01, 0.08; *p* = 0.006; test for endogeneity *p* = 0.001) and psychomotor speed (pegboard dominant hand β = 0.02; 95% CI 0.006, 0.05 test for endogeneity *p* = 0.003; pegboard nondominant hand β = 0.04; 95% CI 0.01, 0.06; test for endogeneity *p* = 0.01)
			Memory	California Verbal Learning Test		
			Attention	Trail Making Test Parts A		
			Executive functioning	Trail Making Test Part B, Clock-drawing Test		
			Phonetic and Semantic Fluency	Letter and Category fluency		
			Confrontation Naming	Naming Test		
			Working memory and Verbal concept formation and reasoning	Digit Span Backwards and Similarities		
			Verbal abilities	The Wide Range Achievement Test Letter and Word Reading subset		
			Psychomotor speed Visuospatial abilities and figural memory	Purdue Pegboard Rey–Osterrieth Complex Figure		
Olsson et al. (2017) [21]	1182 Swedish men, average age 71 years.	Longitudinal	Global Cognition	MMSE	Age and the season of blood collection, BMI, education, physical activity, smoking, diabetes, hypertension, hypercholesterolemia, vitamin D supplements, and alcohol intake	No significant association between serum 25(OH)D and measures of cognition (OR 0.63; 95% CI 0.27–1.48)
			Alzheimer’s Diagnosis	Two physicians completing chart review		
Schneider et al. (2014) [22]	1652 participants, average age 62, 52% White, 60% female	Longitudinal	Verbal learning	Delayed Word Recall Test (DWRT)	Age, gender, education, income, physical activity, smoking, alcohol intake, BMI, waist circumference, and use of vitamin D supplements	No significant association between serum 25(OH)D and measures of cognition. Results reported on each measure for Whites (DWRT: OR 1.09; 95% CI 0.66–1.81; DSST: OR 1.13, 95% CI 0.70–1.84; WFT: OR 1.04, 95% CI 0.65–1.66) and Blacks (DWRT: OR 1.38; 95% CI 0.86–2.23; DSST: OR 0.82, 95% CI 0.50–1.36; WFT: OR 1.11, 95% CI 0.68–1.82)
			Executive Functioning and Processing speed	The Digit Symbol Substitution Test (DSST)		
			Executive functioning and Language	The Word Fluency Test (WFT)		

Abbreviations: mMMSE (Modified Mini-Mental State Exam); BMI (Body Mass Index); MMSE (Mini-Mental State Exam); GDS-K (Korean version Geriatric Depression Scale); CIRS (Cumulative Illness Rating Scale); 3MSE (Modified Mini-Mental State Exam).

**Table 3:** Randomized control trials examining effects of serum 25(OH)D supplementation on cognitive function.

Citation	Sample (Size, Key Characteristics)	Intervention	Cognition Aspect/Measures		Covariates	Outcomes
Dean et al. (2011) [23]	128 healthy young adults with an average age of 21.8 years (SD 2.9), 57% female, 50% Asian	Group A received 5000 IU of cholecalciferol daily and Group B received a placebo daily for six weeks	Visuospatial working memory	N-Back	None	No significant improvements in cognitive functioning (working memory F = 1.09, *p* = 0.30; response inhibition F = 0.82, *p* = 0.37; cognitive flexibility F = 1.37, *p* = 0.24) in the group receiving vitamin D supplementation compared to the group receiving placebo
			Executive functioning	Stop-signal task response inhibition		
			Cognitive flexibility	Set shifting task		
Pettersen et al. (2017) [24]	82 participants from Northern British Columbia, Canada. High dose group with average age of 56.7 years and low dose group with average age of 52.6 years	Participants in high dose group took 4000 IU of cholecalciferol and participants in low dose group took 400 IU of cholecalciferol daily for 18 weeks	Information processing speed	Symbol Digit Modalities Test	Age, education, sex and baseline performance	Participants with insufficient serum 25(OH)D levels at baseline and in the high dose group had significant improvement in pattern recognition memory (Pre M 86.2; SD 14.1; Post M 93.1; SD 7.8, *p* = 0.005). Participants receiving the low dose vitamin D supplementation improved significantly in verbal memory (Pre M 33.7; SD 2.4; Post M 34.6; SD 1.7 *p* = 0.054).
			Executive functioning	Phonemic fluency and One-Touch Stockings of Cambridge		
			Attention and working memory	Digit Span forward and backward		
			Learning/Memory	Verbal Recognition Memory, Pattern recognition, and Paired Associate Learning		
			Working Memory	Spatial Working Memory		

**Table 4:** Key points regarding serum 25(OH)D and cognition.

Observational studies support a significant association between low serum 25(OH)D and compromised cognition, which is further supported by the presence of VDR in neurons and glial cells of the hippocampus, hypothalamus, cortex, and subcortex.
Data from randomized controlled trials is extremely limited, showing no evidence of cognitive improvements after short-term supplementation (4000–5000 IUs daily vs. 0–400 IUs daily) over 6–18 weeks.
The MMSE was the most common cognition instrument used across studies, revealing inconsistent results.
Sunlight, obesity and dietary supplementation are known determinants of serum 25(OH); however, these were not consistently collected or accounted for in analysis across studies
The majority of studies included older participants, therefore it is difficult to make conclusions regarding the role of serum 25(OH)D in cognitive functioning in younger people
Although minority populations are at risk for low serum 25(OH)D, the inclusion of these individuals is extremely limited.

**Table 5:** Key points regarding future research on serum 25(OH)D and cognition.

If feasible, randomized control trials should be conducted.
Power analysis should be completed to determine the necessary sample size to determine significant improvement in cognition
The outcome measure of future research should be cognition measured by a battery of tests (e.g., CANTAB)
The sample should consist of older adults who are at risk for poor cognition and low serum 25(OH)D
The sample should represent minority populations who are at risk for low serum 25(OH)D and poor cognition
